# Understanding human influence on climate change in China

**DOI:** 10.1093/nsr/nwab113

**Published:** 2021-06-29

**Authors:** Ying Sun, Xuebin Zhang, Yihui Ding, Deliang Chen, Dahe Qin, Panmao Zhai

**Affiliations:** National Climate Center, Laboratory for Climate Studies, Beijing 100081, China; Collaborative Innovation Center on Forecast and Evaluation of Meteorological Disasters, Nanjing University of Information Science & Technology, Nanjing 210044, China; Climate Research Division, Environment and Climate Change Canada, Toronto M3H 5T4, Canada; National Climate Center, Laboratory for Climate Studies, Beijing 100081, China; Department of Earth Sciences, University of Gothenburg, Gothenburg 405 30, Sweden; China Meteorological Administration, Beijing 100081, China; State Key Laboratory of Severe Weather, Chinese Academy of Meteorological Sciences, Beijing 100081, China

**Keywords:** human influence, detection and attribution, climate change, climate extremes

## Abstract

China's climate has been warming since the 1950s, with surface air temperature increasing at a rate higher than the global average. Changes in climate have exerted substantial impacts on water resources, agriculture, ecosystems and human health. Attributing past changes to causes provides a scientific foundation for national and international climate policies. Here, we review recent progress in attributing the observed climate changes over past decades in China. Anthropogenic forcings, dominated by greenhouse gas emissions, are the main drivers for observed increases in mean and extreme temperatures. Evidence of the effect of anthropogenic forcings on precipitation is emerging. Human influence has increased the probability of extreme heat events, and has likely changed the occurrence probabilities for some heavy precipitation events. The way a specific attribution question is posed and the conditions under which the question is addressed present persistent challenges for appropriately communicating attribution results to non-specialists.

## Teaser

Human influence on China's climate is clear. Anthropogenic forcing is the main driver of the observed warming in mean and extreme temperatures, and its effect on heavy precipitation is emerging.

## INTRODUCTION

**Figure 1.**
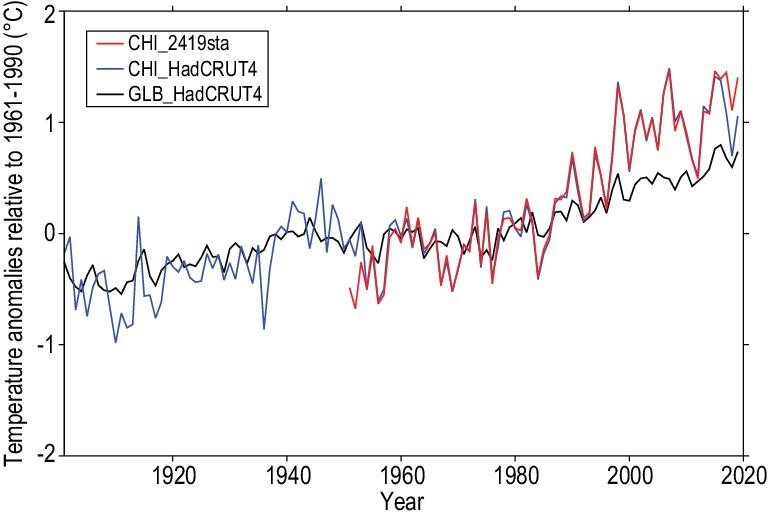
China's surface air temperature has increased at a greater rate than the global mean surface temperature. Clear trends can be seen in the time series for the global (black) and regional (over China) mean (blue) surface temperature in the HadCRUT4 datasets for the period 1901–2018, and also in the regional average (red) calculated from homogenized data at 2419 Chinese observing stations for 1951–2018. Temperature is expressed as the anomaly (°C) relative to respective 1961–1990 averages.

Human activity, dominated by the emission of greenhouse gases, has resulted in an increase of about 1.0°C in the average Earth surface air temperature since pre-industrial times [1]. The warming has affected all parts of the climate system, including the atmosphere, ocean, land, cryosphere and biosphere. On a regional scale, surface air temperature over China has warmed at a rate much higher than the global average (Fig. 1) [2]. As the warming continues, its impacts on human and natural systems increase; and global warming becomes the most challenging problem for the world. Central to climate change policy is an understanding of the causes of past climate change, at both global and regional scales.

Since the late 1980s, the successive assessments of the Intergovernmental Panel on Climate Change (IPCC) have established that human influence has resulted in global warming, mainly through the emission of greenhouse gases. When each assessment report was released, it prompted a new set of international climate treaties and/or policies. The first IPCC report, published in 1990, identified that human use of fossil fuels had substantially increased the concentration of atmospheric greenhouse gases, leading to an enhanced warming effect and resulting in a warming of the Earth's surface [3]. This report brought climate change to the attention of international politics for the first time, serving as the basis for the founding of the United Nations Framework Convention on Climate Change (UNFCCC) in 1992. The release of the second IPCC assessment report in 1996 [4] confirmed that global warming was ‘unlikely to be entirely caused by nature’ and that human activities have had a ‘discernable’ impact on the global climate system, resulting in the adoption of the ‘Kyoto Protocol’. The third IPCC assessment report [5], published in 2001, provided a scientific consensus that ‘most of the warming observed over the last 50 years is attributable to human activities’. This led to the inclusion of both adaptation and mitigation in the UNFCCC negotiations. The fourth IPCC assessment report, released in 2007, concluded that the ‘warming of the climate system is unequivocal’ and that most of the warming observed since the mid-20th century ‘is very likely due to the observed increase in anthropogenic greenhouse gas concentrations’ [6]. The conclusions of this report were the scientific basis for the UNFCCC’s ‘Bali Road Map’. The fifth IPCC Assessment was completed in 2014, stating that ‘human influence on the climate system is clear’ [7], providing the science foundation for the ‘Paris Agreement’. This Paris Agreement aims at holding the increase in the global average temperature to well below 2°C above pre-industrial levels and to pursue efforts to limit the temperature increase to 1.5°C above pre-industrial levels. Our understanding of the causes of climate change along with the confidence in this understanding was key in each of these assessments and the international climate policies that they informed.

While international climate policies have significant implications for national policy, understanding regional climate changes is also highly relevant for national climate policymaking as policymakers can relate more directly to climate change at national and regional scales. For this reason, there has been a concerted effort to systematically assess climate change in China since the late 1990s. This effort has resulted in the production of three national climate change assessments [8] and three national science assessment reports on climate and environment changes [9]. Literature on attributing the observed changes in the climate in China to specific causes available to these assessments is limited because research in this field began relatively late in China. However, significant advances have been made in recent years, thanks to the increasing interest of Chinese climate scientists in this subject, and the increasing levels of support from various funding agencies. We review these recent advances here, focusing on the human influences on China's climate including detection and attribution of long-term changes, and the attribution of changes in the frequency and/or magnitude of high impact climate events. Our review is built on earlier general review papers [10–13], focusing on China to inform ongoing and future national assessments. We also cover new development since those earlier reviews. The paper is organized as follows: we first describe climate change detection and attribution methods to provide a general context for the interpretation of attribution results; we then review the attribution for long-term changes in various variables and recent developments in the field of event attribution; finally, we provide conclusions and suggested directions for future research.

## METHODS OF ATTRIBUTION

‘Attribution’ implies determining the relative importance of different drivers behind a change. The drivers can be both internal to the climate system or external forcing. Internal factors include decadal and multi-decadal natural climate variability. External forcings can be anthropogenic such as greenhouse gas and aerosol emissions or land-use change [6,7], or natural such as volcanic activity. An attribution usually involves collection and quality control of observation data, identification of possible drivers and causality inferencing. There are two types of attribution studies in the climate literature. One deals with the attribution of long-term changes in the mean climate and in climate extremes; the other focuses on the changes in the magnitude or frequency of specific extreme weather and climate events. Methods for both types of study have evolved but the length constraint of this paper prohibits a detailed and comprehensive review of the methodologies. Here, we aim at providing a conceptual framework for methods that are popular in the recent literature and highlighting issues that are relevant to the proper interpretation of the attribution studies.

### Long-term climate changes

The objective of most detection and attribution studies for long-term changes in the climate is to determine whether changes have occurred, and if so, to attribute them to specific causes. Regression-based methods, also known as fingerprinting methods, have been widely used. Most of the detections and attribution studies reviewed in this paper use the optimal fingerprinting method [14–17]; for this reason, we will provide a conceptual introduction and discuss its limitations here. This method regresses observations onto an expected climate response simulated by climate models under different external forcings. The method can be traced back to Hasselmann [18], who coined the term ‘optimal fingerprints’. The link between ‘optimal fingerprints’ and linear regression was made explicit by Allen and Tett [19]. In a nutshell, this method assumes that climate models can correctly simulate the patterns of the climate response to external forcings, even though the magnitudes of the response patterns may differ from the observations. With this assumption, it is possible to regress observations onto model-simulated responses, and detection and attribution analysis is reduced to statistical inferencing about the regression coefficients or scaling factors. The analysis requires three main ingredients: (a) observation data with sufficient spatial and temporal coverage and high quality; (b) climate-model-simulated responses to one or more external forcings, typically estimated as the ensemble averages from multi-model simulations; and (c) estimates of internal variability of the climate which are required for solving the regression problem, and for estimating the uncertainty in the scaling factors and thus making statistical inferences. Internal variability is typically estimated from a large number of pre-industrial climate simulations. Detection of the climate response to a particular external forcing can be claimed if the corresponding scaling factor is significantly above zero, and attribution can be claimed if the confidence interval also includes one and if influences from other external forcings can be excluded.

Regression models with various levels of complexity have been used, depending on how uncertainty is treated for the observations, and the responses simulated by the models. The simplest form of regression, termed ordinary least squares [19], does not consider uncertainty in the observation or in the model-simulated responses. The uncertainty in the estimated responses because of internal variability tends to bias the best estimates for the scaling factors towards smaller values, and underestimates the uncertainty associated with the scaling factors. Consequently, most detection and attribution analyses use the total least squares method (TLS, [14]), which is a more complex model that explicitly accounts for the uncertainty in the estimated responses. Although climate modelling centers have produced large volumes of pre-industrial climate simulations, the availability of these control-run data is still too small for robust estimation of the covariance if the detection and attribution analysis is implemented at high spatial and/or temporal resolution. Additionally, when the amount of simulation data is too small, the covariance matrix may not be full rank, and may therefore not be invertible. Dimension reduction and the use of regularized covariance [15] have been used to circumvent this problem. While methods exist that consider multiple sources of uncertainty for estimated model responses, imperfect measurement of the observations and internal variability [20–22] mean that their applications have been limited.

Generalized multivariate regression assumes that the regression residuals follow a multivariate Gaussian distribution. This assumption is often justified for means of climate variables. Direct implementation of the optimal fingerprinting method for climate extremes may be problematic because extreme values, such as the annual maximum daily precipitation at a location, would be skewed, and would generally follow extreme value distributions. Two approaches have been used to take into account the distributional property of extreme values, or of indices for climate extremes, in detection and attribution analyses for long-term changes in climate extremes. One approach is to convert the extreme values, such that the new quantities are not skewed, and the distributional assumption of optimal fingerprinting method is satisfied. Two methods have been used for this. One method converts the original observation data into a probability-based index [23–28]. This probability transfer makes it possible to use the optimal fingerprinting method, and makes it easier to compare extreme values at different locations, but the results can be difficult to interpret physically. The second method averages the extreme values over a large region, so that the averages asymptotically approach a Gaussian distribution [29]. A caveat to this method is that it is not always meaningful to average extreme values, for example, extreme precipitation over a large region that encompasses diverse climate conditions and within which observations are unevenly distributed. An alternative approach is to explicitly fit the observations to extreme value distributions that are constructed from the model-simulated responses, as covariates of the distribution parameters [30–32]. A potential problem with this method is that it is difficult to account for the uncertainty in a single estimate.

Specific regional conditions complicate the attribution of regional-scale climate change. For example, urbanization or other land-use changes can enhance or counteract greenhouse gas effects, and local aerosol forcings can play a significant role on the regional scale. Yet these forcings are often not considered in climate simulations, or are poorly represented, making it difficult, and sometimes impossible, to estimate the pattern of the climate response to the applied external forcing. Climate variability is also greater at smaller spatial scales, making it more difficult to identify regional climate change than global climate change.

### Extreme events

Most damage from natural disasters is related to extreme weather and climate. With increased awareness of global warming and an increase in loss and damage, the media and the public often ask whether the human influence on the climate system has caused specific high-impact weather and climate events, such as the 2003 European heatwave and the 2013 hot summer in eastern China during or after the event. New methods have been developed to address such questions, albeit often indirectly. Since the pioneering work of Stott *et al.* [33], event attribution has emerged as a distinct field of science [12,34,35]. It is now possible to estimate the human contribution to changes in the probability for the occurrence of, or magnitude of, such events.

Very few studies attribute a particular event [36], and few studies attempt to conduct ‘end-to-end’ attribution, which is attribution for the impacts of an extreme event [37]. Some recent developments in event attributions rely on process understanding, following what is called ‘a story-line approach’ [38]. However, most event attribution studies focus on a specific class of events and ask whether human influence has affected the probability and/or magnitude of events in that class. For example, while the 2013 summer heatwaves in eastern China may have motivated an event attribution study, the question that is often asked relates to events that are similar, in other words, to the class of events that are similar to the 2013 summer heatwaves. These studies almost always involve a comparison between the magnitudes or probabilities for the event in the factual world (the world that has been), and in the counterfactual world (the world that might have been, had we not emitted greenhouse gases since pre-industrial times). Approaches to these comparisons differ depending on how the factual and counterfactual worlds are constructed, and how the questions are asked [12]. For example, probabilities for an event occurring in both worlds are often estimated to construct the so-called fractional attributable risk (FAR), or risk ratio (RR), which then determines the level of human influence on the event. Estimates of FAR or RR can be quite different if the problem is framed differently, for example, if different metrics are used to describe a particular variable, and/or for the definition, magnitude and rarity of an extreme event, and/or for its spatial and temporal extent [39]. The observation data and climate models, and the method used to estimate the probability, all affect the attribution results.

The conditioning used to simulate the factual and counterfactual worlds is important. When coupled-climate-model simulations are used in event attribution analysis, the only required condition is the external forcing. It is therefore possible to estimate changes that are the result of specific external forcing, for example the emission of carbon dioxide. When simulations from atmosphere-only models are used, the simulations are conditional to the observed patterns of sea surface temperature, and sometimes a particular configuration of circulation patterns, in addition to the specific external forcing. Thus, attribution of a model response to the specific external forcing is conditional on the particular sea surface temperature or circulation patterns that were used as conditioning for the simulations. As it is not possible to estimate the probability for any particular circulation configuration, it is also not possible to absolutely determine the effect of human influences. Event attribution results should therefore always be interpreted in the context of how the problem was framed, and of the conditioning used for the simulation. This makes it difficult to synthesize results from different studies and to communicate findings to non-specialists. However, conditional estimates do provide means for a story-line approach to explain attribution.

Lack of verification is an important caveat to event attribution methodologies. The counterfactual world, the world that ‘would have been’, had human influence not existed, is not observable. Thus, it is not possible to know to what extent the model has faithfully simulated that counterfactual world. More importantly, the events that are under investigation typically have a very small probability of occurrence in the factual world, and perhaps an even smaller probability of occurrence in the counterfactual world. This makes it difficult to evaluate the accuracy of the model-simulated probability, or magnitude, for the event in the counterfactual world.

## DETECTION AND ATTRIBUTION OF LONG-TERM CLIMATE CHANGE

The annual mean near-surface air temperature over China has increased rapidly since the mid-20th century (Fig. 1). The rate of warming was about 0.24°C/decade during 1951–2019 [2], which is greater than the global average (∼0.12°C/decade), and the global land average (∼0.18°C/decade) during 1951–2012 [7]. The strongest warming was observed in northern China in the winter. The rapid increase in the mean temperature was accompanied by changes in climate extremes, in moisture levels and in the wet bulb globe temperature in summer [2,40]. A large body of literature has emerged with the aim of understanding the causes of these observed changes.

### Surface air temperature

Attribution for long-term temperature changes has been based mainly on the optimal fingerprinting method [15,19], using homogenized station data from China and climate simulations from the Coupled Model Intercomparison Project, Phases 3 and 5 and more recently Phase 6 (CMIP3, CMIP5 and CMIP6 [41–43]). These studies consistently find that anthropogenic influence made an important contribution to the rapid warming.

**Figure 2.**
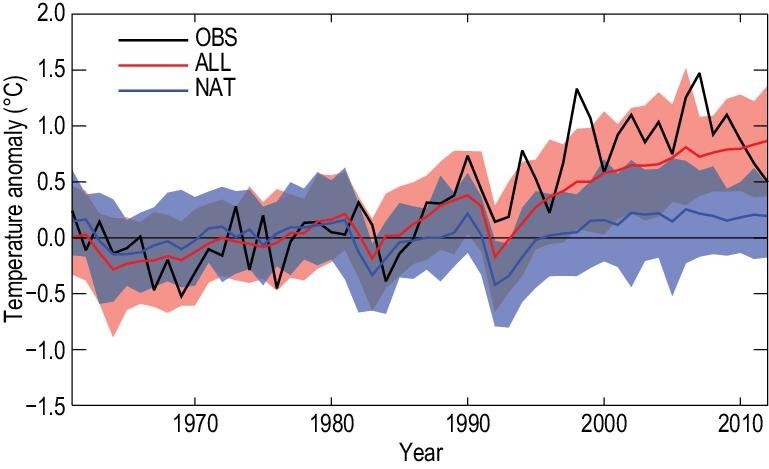
The observed change in mean temperature (OBS, based on Chinese 2419 station data) is consistent with the CMIP5 model-simulated change when the combined effects of all external forcings are included (ALL), but is not consistent with model simulations when only natural external forcings (NAT) are considered. The figure shows the mean temperature anomaly (°C, relative to the 1961–1990 average) during 1958–2012. Shading indicates the 5–95% ranges for the simulated responses under the ALL (red) and NAT (blue) forcings, with the overlap in the range shown as dark purple. Adapted with permission from Ref. [46]. Copyright 2016 Nature Publishing Group.

**Figure 3.**
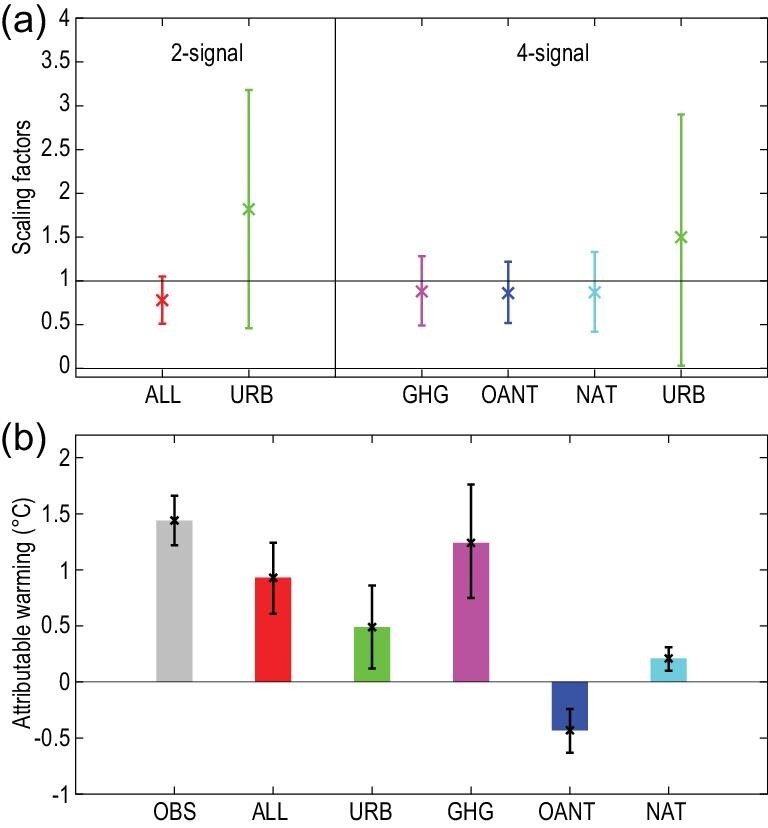
The signals including ALL, GHG, OANT and urbanization effects (URB) can be detected in the observed mean temperature changes in China since the late 1950s. (a) The best estimates of the scaling factors that scale the signals to match the observed temperature anomalies and their 5–95% uncertainty ranges, for the ALL and URB signal patterns in the two-signal analysis shown on the left and the URB, GHG, OANT and NAT signal patterns in the four-signal analysis shown on the right. (b) Best estimates of the observed annual mean temperature trend and its attribution to ALL and URB from the two-signal analysis, and to GHG, OANT and NAT from the four-signal analysis, along with their 5–95% uncertainty ranges. Adapted with permission from Ref. [46]. Copyright 2016 Nature Publishing Group.

Earlier studies have detected the effect of combined greenhouse gases and sulfate aerosols on the observed warming [44,45]. Sun *et al.* [46] expanded on the earlier studies, and were the first to consider, simultaneously, all known drivers for surface temperature change, including both external natural and anthropogenic forcings (ALL forcing) to the climate system, and the local and regional effects of urbanization. Figure 2 shows that the CMIP5 model forced with the ALL forcing reproduced the observed temperature increase in China, with a slight underestimation mostly related to urban heat island effects. Using the optimal fingerprinting method, the contributions from four drivers were quantified and separated: greenhouse gases (GHG), other anthropogenic factors (OANT) including anthropogenic aerosols and changes to land cover and land use, natural external forcings (NAT) including solar and volcanic forcings, and urbanization (URB). Figure 3 shows that the mean temperature increased by 1.44°C (90% confidence interval: 1.22–1.66°C) during 1961–2013. Two-thirds of the warming, 0.93°C (0.61–1.24°C), can be explained by the combined influence from the ALL forcing on the global climate system, which is similar to the observed warming in global land mean temperature during 1951–2010. GHG alone may have contributed 1.24°C (0.75–1.76°C) to the warming, 35% of which may have been offset by the cooling effects of OANT (OANT is dominated by aerosols). The contribution of the cooling effect is also similar to that of global land temperatures. The NAT forcings have contributed 0.21°C (0.10–0.31°C) to the warming, although the reliability of this estimate may be affected by underestimation of the volcanic forcings for the CMIP5 simulations [47]. The remaining one-third of the observed warming, 0.49°C (0.12–0.86°C), was explained by urbanization effects. Two different analyses were used to estimate URB contribution and both produced similar results, indicating the robustness of the estimations. The best estimate shows a large contribution from urbanization, but there is a large uncertainty associated with this estimate, which is consistent with observation-based estimates [48,49]. In another study, Zhao *et al.* [50] showed that GHG-induced warming was three times that of the observed warming, offset by a large aerosol cooling of a magnitude about 1.5 times that of the observed warming. The qualitative conclusion that GHG forcing is the main contributor to the observed warming, offset by the aerosol cooling effect, is consistent with results from other studies. But the quantitative results are unlikely to be realistic and are possibly an artifact of regression degeneracy because of dependency between GHG and aerosol signals that violates the independence assumption of the regression method used in the study. Anthropogenic influences on temperature also can be detected at seasonal and/or sub-country scales. For example, they contributed more than 90% of the summer warming in eastern China during 1955–2013 [51] and are the dominant factor for the increases in annual mean temperatures in western China [52].

In addition to attribution for the mean temperature, a recent study attributed changes in an impact-relevant heat stress indicator, the wet bulb globe temperature (WBGT). WBGT takes into account both dry air temperature and humidity, and is widely used to reflect heat stress, which affects the ability of the human body to dissipate excess metabolic heat. Li *et al.* [40] examined possible human influences on the observed changes in WBGT and their contribution to China's record-high summer WBGT. They showed that the observed changes in summer mean WBGT in China since 1961 were consistent with the model-simulated response when ALL forcings were used. WBGT increased 1.17°C in western China and 0.70°C in eastern China during 1961–2010, and there was less than 1% chance that such an increase could occur without human influence. The occurrence of the highest summer WBGT during 1961–2015 has become more than 1000 times as likely in western China, and more than 140 times as likely in eastern China during 2011–2020 than it was during the 1961–1990 baseline period.

### Precipitation and atmospheric moisture

Precipitation response to anthropogenic forcing is projected to be spatially variable, with a large percentage increase in high latitudes and a decrease in dry mid-latitude regions, and an increase in moist mid-latitude regions by the end of the 21st century under the RCP8.5 scenario [53]. This implies that anthropogenically induced changes in total precipitation over China could have been small so far and would be difficult to detect because China is geographically located in the transition zones between the region with a projected increase and the region with a projected decrease in precipitation. There has not been a clear precipitation trend over China as a whole since systematic observation began in the 1960s. At a sub-country scale, observations show an increase in southern China and a decrease in northern China over recent decades [54,55]. These opposing trends are the opposite of the expected precipitation response simulated by climate models [56]. There is a range of explanations for the observed changes, including the effects of aerosols, changes in the monsoon circulation system and in sea surface temperature in the Pacific, the Indian and the Atlantic Oceans [57,58].

In a warmer world, the specific humidity of the atmosphere increases but the relative humidity over land remains unchanged, or decreases slightly [7]. Zhang *et al.* [59] found that this was the case for China: the total column moisture, or precipitable water, increased during 1973–2012 and the increase was mainly a result of anthropogenic influence. The increase in precipitable water is consistent with warming but it is unclear how the changes in moisture affect precipitation as atmospheric circulation plays an important role and the circulation response to external forcings is still largely unknown for this region.

### Extreme temperature

Various indicators of temperature extremes have shown warming over China since the late 1950s [8], consistent with warming in mean temperature. In most regions, warm extremes have become more intense and more frequent, and have lasted longer; while cold extremes have become less intense and less frequent, and are more short-lived than previously. The length of the growing season has increased, and the numbers of frost days and ice days have decreased. Hot extremes, such as the number of summer days and tropical nights, have increased. There are a few exceptions, such as the cooling features seen in southwestern China [8,60]. Recent studies have consistently shown that human activities contributed to these changes.

**Figure 4.**
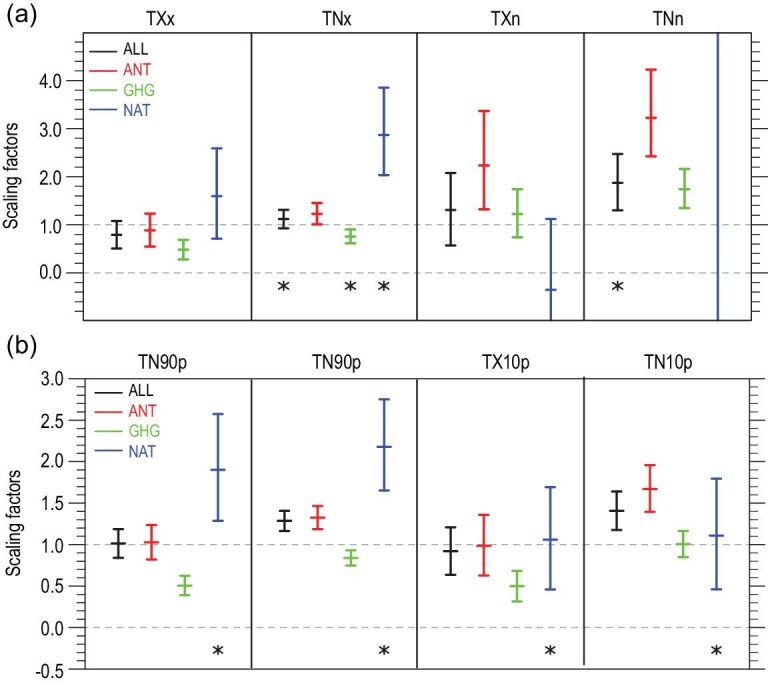
Human influence can be detected in the changes in the intensity (TXx, TNx, TXn and TNn) and the frequency (TX90p, TN90p, TX10p and TN10p) indices of temperature extremes. (a) The best estimates of the scaling factors and their 5–95% confidence intervals for ALL, ANT, GHG, and NAT in one-signal detection analyses for four annual series of intensity indices over China for the period 1958–2012. An asterisk indicates higher variability in the model simulations according to the residual consistency test. (b) As (a) but for the percentile frequency indices. Adapted with permission from Refs. [61] and [62]. Copyright 2017 John Wiley & Sons publications and 2016 American Geophysical Union.

Wen *et al.* [29] were the first to provide clear evidence of human influence on the intensity of extreme temperatures, including the annual maximum and minimum temperatures during the day and night, in China. Based on simulations by one climate model, they detected a human contribution to changes in extreme temperatures, and were able to separate the anthropogenic forcing signal from the natural forcing signal. Yin *et al.* [61] significantly improved upon Wen *et al.* [29], using a newer and more up-to-date detection and attribution technique with a multi-model ensemble of climate simulations and a longer time series of observational data. This study confirmed that the earlier findings were robust, and showed that the anthropogenic signal could be clearly and robustly detected in the intensity of extreme temperature events (Fig. 4a).

There is also clear and robust evidence of human influence on the frequency of extreme temperatures represented by the number of cold and warm days and nights (Fig. 4b, from Ref. [62]), the duration of warm and cold spells [63], or the number of frost days, tropical nights, ice days and summer days [64]. A more recent study [65] used simulations from the newest CMIP6 models and updated observations (HadEX3 data [66]), and confirmed the contribution of human influence to the frequency of temperature extremes in Asia, including China. This newer study successfully separated the influence of greenhouse gases from that of aerosols, enhancing confidence in the attribution. In addition to global warming influences, the effect of local urbanization on extreme temperature indices for eastern China was also detectable [67].

Human influence can be detected in the frequency and intensity indicators of temperature extremes at the sub-country scale. This includes detection in eastern and western China [61,62]. Additionally, Yin *et al.* [68] identified the effects of human influence in a set of 12 extreme temperature indices for the Tibetan Plateau during 1958–2017, with greenhouse gas emissions playing the dominant role. Compared with changes to temperature extremes over China as a whole, changes to temperature extremes on the Tibetan Plateau have occurred at a higher rate, which is consistent with the relatively stronger warming experienced in this region.

### Extreme precipitation

A decrease in light precipitation and an increase in heavy precipitation have been detected in parts of China, especially in eastern China [69–71]. There is some consistency between these observed changes in eastern China and other regions of similar latitudes, leading to the speculation that global warming may have played a role [70]. However, there is also evidence suggesting that anthropogenic aerosols may be closely linked to such changes [70,72]. The urbanization effect on the heavy precipitation in cities has also been suggested [73]. No formal attribution study for light precipitation has been conducted so far. Thus, in what follows the focus is placed on heavy precipitation.

Observed changes in heavy precipitation in the second half of the 20th century over China are spatially non-uniform. Heavy precipitation has increased in eastern China and light precipitation has decreased [8,74]. Yin and Sun [75] studied precipitation data ending in 2017, and found increasing trends in several indices for precipitation extremes, particularly in those used to indicate heavy precipitation. Several studies have examined possible human influences on extreme precipitation, using different metrics and different methods of analysis [76–81]. Some have identified the effects of human influence on extreme precipitation, with various levels of robustness, and some have not. In general, these studies point to the emergence of the detectable effects of human influence on extreme precipitation, although the attribution is not as robust as for extreme temperature.

**Figure 5.**
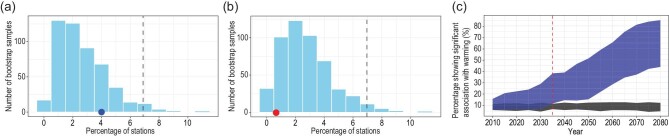
The observed increase in annual maximum daily precipitation is not significantly different from what could be expected by chance, but an increase can be robustly detectable by the mid-2030s. (a) and (b) Number of bootstrap samples showing the percentages of stations in China with a significant increase (a) or decrease (b) trend in extreme precipitation during the period 1961–2012 in the 500 bootstrap samples. The dashed lines mark the percentage corresponding to the 95th percentile of the probability distribution from the bootstrap samples. The solid circles on the horizontal axis show percentage of stations with significant trends in the original non-permuted station data, both are below the 95th percentile of the bootstrap samples. (c) Fraction of CMIP5 model grid boxes over China that have significant association between annual maximum daily precipitation and the global mean near-surface temperature anomalies at the 5% level. Blue shows model spread for the simulations forced with the RCP 8.5 (Representative Concentration Pathway 8.5) scenario, while gray shows expectations by chance based on 200 bootstrap samples at the 95% level. The red vertical dashed line indicates the time when an anthropogenic influence on extreme precipitation is detectable in simulations of models. Adapted with permission from Ref. [77]. Copyright 2018 American Geophysical Union.

One set of metrics is the annual maximum precipitation amounts that fall in a single day (Rx1day), and over five consecutive days (Rx5day). These quantities are used in a wide range of applications, including engineering design, so understanding how they may change is important. At present, the most robust evidence of human influence on these quantities is at a hemispheric scale, for land regions where observation data are more abundant [23,25]. Li *et al.* [76] applied the method from Min *et al.* [23] and Zhang *et al.* [25] to study changes in the Rx1day and Rx5day in China, by transforming the data to probability-based indices, and then comparing the observations based on gridded precipitation datasets with CMIP5 simulations. They were able to detect an anthropogenic signal, but the signal from ALL forcings, which includes both anthropogenic and natural forcings, was surprisingly not detected. The lack of detection of an ALL forcing signal suggests that the detection of the anthropogenic signal may not be robust as a response to ALL forcing should be closer to observations than that to anthropogenic forcing alone. Li *et al.* [77] also examined changes in Rx1day in China, but used a different method. They fitted the observed Rx1day data to a generalized extreme value distribution with global mean temperature as a covariate. The detection of the global warming signal is based on a field significance test: a signal is considered to have been detected if the proportion of stations with Rx1day significantly correlated to global mean temperature is larger than would be expected by chance (Fig. 5). They found that the global warming signal was not detectable in the observed Rx1day data by the year 2012, but the signal could have been robustly detected by the 2030s in a perfect model setting based on CMIP5 simulations. While the conclusions from these two studies appear to be inconsistent, they both indicate that an anthropogenic signal is emerging in Rx1day, although detection may not be very robust at this time. This finding seems to be supported by Ma *et al.* [78], who showed that anthropogenic influence may have shifted the probability distribution for daily precipitation

towards more heavy precipitation in eastern China. This is also supported by Chen *et al.* [79], who showed that daily and hourly extreme precipitation intensified in eastern China during 1970–2017, and that the intensification can be explained by an increase in the global mean temperature.

The frequency of extreme precipitation is also used as a metric. Chen and Sun [80] defined extreme precipitation events as daily precipitation that occurs once in three or ten years during 1960–2014. They found that the nationally aggregated frequencies for these two types of extreme precipitation events increased between 1960–1979 and 1980–2014 based on 542 long-term stations in China. A similar increase was found in the CMIP5 simulations under ALL forcing but not in those under natural forcings. The similarity between the observed changes in extreme precipitation frequency and that in the ALL forcing simulations suggests a possible human influence on extreme precipitation. However, this finding in itself is insufficient for attribution of the observed changes to external forcings, as a direct causal link cannot be established. Chen and Sun [80] also used an optimal fingerprinting approach to compare nationally averaged frequencies in the observations and in the CMIP5 simulations.

The third set of metrics is the annual amount of precipitation falling during days of heavy precipitation, expressed either as an absolute amount, or as a proportion of the total annual precipitation, as defined by the Expert Team on Climate Change Detection and Indices (ETCCDI) [81]. Here, heavy precipitation days are defined as days when the daily precipitation exceeds the 99th or 95th percentiles of wet-days, from a distribution calculated for a base period. Dong *et al.* [82,83] compared these quantities over Asia, in the observations and in the CMIP5 and CMIP6 simulations, using the optimal fingerprint method. They found an anthropogenic signal in those quantities for mid-latitude Asia. As most of the stations used in the studies are located in China, this may indicate a detectable human influence on this metric over China.

## ATTRIBUTION OF EXTREME EVENTS

### High temperature

Warming in China over recent decades comes with an increase in extreme high-temperature events. In particular, all five of the hottest summers in eastern China since the 1950s occurred in the 21st century [51]. The occurrence of such events, which often broke historical records, has sparked a significant effort in China to study possible human influence on their magnitude and frequency. Since the publication by Sun *et al.* [51] on the 2013 extreme hot summer in eastern China, many studies have investigated the human influence on high-temperature events in different regions of China. These studies have used different analytical methods including simulations from atmospheric-only or coupled climate models, different metrics including the number of warm spring days [84], the number of summer heatwave days [85], the maxima for daily maximum and minimum temperatures [86] and the number of consecutive high-temperature events [87]. These studies consistently show that anthropogenic forcings have substantially increased the probability of high-temperature events in China. While different factors including atmospheric circulation [88], sea surface temperature [89] and the effect of urbanization [90] have been considered, anthropogenic forcings appear to have played the dominant role in the increase in the magnitude and frequency of high-temperature events.

Coupled-model simulations have been widely used in these studies. Sun *et al.* [51] were the first to use the coupled model simulation when analyzing human influence on the 2013 summer heatwaves in eastern China. Rather than directly estimating the effects of human influence on heatwaves, which is difficult to do and is also sensitive to the definition of heatwaves, Sun *et al.* [51] analyzed human influence on 2013 summer mean temperatures. They first established that summer mean temperature and heat wave metrics are closely linked, and that summer mean temperature can be used as a proxy for summer heatwaves. Then, they used an optimal fingerprint approach to compare the observed and CMIP5-model-simulated summer mean temperatures, and found that the observed change in summer mean temperature can be attributed to human influence. They finally reconstructed the human influence on summer mean temperature to correct bias in climate model simulations and to estimate the occurrence probabilities for 2013-summer-like temperatures in a model world, both with and without human influence. Because variability in model simulations is validated with observations, and because the model bias is corrected, the probability ratio should be quite reliable when calculated this way. This approach was later adopted in several other studies, including Song *et al.* [84] who looked at the warm spring in northern China in 2014, Sun *et al.* [86] who studied high temperatures in western China in 2015 and Li *et al.* [40] who examined the effects of human influence on hottest WBGT. Miao *et al.* [91] applied a similar but different method to study human contribution to the record-breaking temperature in northwest China in July 2015, involving coupled model simulations, and found that human influence had increased the probability of the event three-fold. Zhou *et al.* [90] considered the effects of warming-induced thermodynamic and dynamic changes, as well as urbanization, and found that they all contributed to the 2018 record-breaking summer heat in northeastern China.

Several studies have relied on atmosphere model simulations to estimate the effects of human influence on extreme heat. Typically, these models have been driven by observed sea surface temperature (SST) and sea ice to simulate the world as it has been, and by SST and sea ice conditions with the effect of global warming removed to simulate the world as it would have been without human influence. Some studies use large-ensemble simulations conducted with the HadGEM3-A model because of the availability of the simulations and their near real-time updates [92]. For example, Chen *et al.* [85] analyzed July 2017-like heat waves over central-eastern China. They found the event to be a one-in-five year event, but this would have been rare in a world without human influence.

While simulations from both coupled and atmosphere-only models have been used for event attribution, and results can be quite similar, it is important to stress again the differences between the two for results to be interpreted appropriately. Simulations from atmosphere-only models are conditional on the observed spatial-temporal patterns of sea surface temperature and sea ice, and thus the occurrence probabilities are conditional on the historical state of atmosphere-ocean variability. As it is not possible to estimate the probability of the particular path that atmosphere-ocean variability has experienced, it is also not possible to estimate the relevant unconditional probabilities, and thus not possible to determine the corresponding probability ratio. Coupled-model simulations are driven by external forcings only, and are not constrained by observed SST and sea ice. As such, there can be a large bias in both the mean and the variability for a particular region. Such a bias needs to be carefully considered. The bias can be adjusted if an optimal fingerprint approach is used, such as in Sun *et al.* [51]. Confidence in probability estimates is high if observed long-term changes can be attributed to human influence. There may be a large difference in event attribution statement based on these two approaches. For example, Sun *et al.* [93] demonstrated that the use of AMIP-type and CMIP-type simulations can lead to large differences in probability estimates.

### Cold event

Warming has resulted in a reduction in extreme cold events in China. Fewer events also mean less attention directed at them, but the 21–25 January 2016 cold event caused widespread impacts including snowfall in Guangzhou and provided an opportunity to closely investigate the human influence on cold events. Three papers have studied this event so far. Two concluded that human influence had made such events less likely to occur [94,95], but the other study suggested that probabilities of extreme circulation anomalies underlying the extreme cold surge have increased because of human-induced Arctic warming [96].

**Figure 6.**
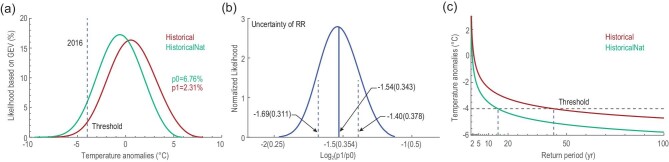
Warming has resulted in a decrease in the occurrence probability of a 2016-like cold surge. (a) The GEV distributions fitted to anomalies of pentad mean daily minimum temperature (Tmin) (°C) during midwinter 2016 averaged over eastern China of historical simulations forced with anthropogenic and natural forcings and historicalNat simulations forced with natural forcings. A shift in the probability distribution because of warming is clearly seen. The dashed line indicates regional average pentad Tmin anomaly for 21–25 Jan 2016 in the observations. (b) Uncertainty in the attributable risk ratio with dashed lines marking one standard deviation. (c) Return period (years) for an extreme cold event with an intensity equal to, or greater than the Jan 2016 event in historical, and in historicalNAT simulations. The black dashed line shows the anomaly of the event and the dashed blue lines represent the return periods of the event in the world with (the line to the right) and without (the line to the left) human influence. Adapted with permission from Ref. [95]. Copyright 2018 American Meteorological Society.

Sun *et al.* [94] analyzed the event using an approach similar to that of Sun *et al.* [51], finding that winter mean temperature had increased as a result of human influence and that the occurrence probability for the 2015/2016 winter-like extreme temperature decreased by about 89% for northern China, and by about 69% for southern China. Qian *et al.* [95] took a quite different approach by estimating occurrence probability for similar events in a world with or without human influence. They fitted generalized extreme value (GEV) distributions to pentad mean daily temperature anomalies for the dates of the event in the simulations conducted with an atmospheric model (HadGEM3-A) that were forced with or without human influence. It is clear from their analysis that the probability distributions of such events shifted to the left in the world with human influence, indicating a reduction in the occurrence of probability for such events (Fig. 6). They concluded that human influence may have reduced the occurrence probability for the 2016 cold event by about two-thirds. Ma and Zhu [96] compared the circulation regime underlying the cold event in the simulations conducted with the MIROC5 model that mimicked the factual world and a counterfactual world. They showed that the occurrence probabilities of circulation anomalies underlying the cold event have increased in response to anthropogenic forcings. They further speculated that the January 2016 cold surge could be a result of global warming. However, it is difficult to validate such speculation because changes in circulation do not necessarily translate into a similar change in the occurrence probability of the cold event. For example, while internally generated circulation anomalies alone could have produced the 2010 Russian heatwave of the magnitude as observed, there was a large 80% probability for the heatwave to not have occurred without human influence [97].

### Heavy precipitation

Event attribution studies of heavy precipitation have used different metrics including amount, frequency, intensity and duration. As different studies have focused on events that occurred in different regions and at different times, and have produced wide-ranging results from strongly attributing heavy precipitation events to human influence to natural variability, it has been difficult to synthesize results from these studies.

Several studies have used simulations by coupled models [98–100]. Sun *et al.* [100] focused on the heaviest June precipitation in South China, and showed that anthropogenic influence had doubled the probability of 2017-like heavy precipitation in southeastern China. Sun and Miao [98] and Yuan *et al.* [99] found large contributions from El Niño and from anthropogenic influences to the extreme precipitation events they studied. Burke *et al.* [101] used HadGEM3-A simulations to investigate the effects of human influence on the duration, amount and intensity of consecutive wet events in May 2015 in 12 sub-regions of southeast China. They found an anthropogenic signal in three out of the 12 regions and concluded that anthropogenically induced climate change has increased the probability of short-duration, intense rainfall events in parts of southeast China. However, Li *et al.* [102] concluded that the strong El Niño in 2015 may have increased the occurrence probability for the rainfall events rather than these being driven by anthropogenic forcing, despite analyzing the same set of model simulations. Zhang *et al.* [103] used simulations conducted with the same model and showed that anthropogenic influence increased the occurrence probability for highest daily precipitation but not for persistent heavy rainfall for the 2018 heavy-precipitation event in central-western China. Based on a set of CMIP5 model simulations including those of HadGEM3, the July 2016 heavy precipitation event over Wuhan was attributed to human-induced warming and El Niño [104].

Heavy snowfall as an important form of heavy precipitation has shown an increase in parts of northern China in the recent decade [105]. These changes seem to be linked to changes in atmospheric circulation [106], but the causes of the circulation change are unclear.

### Drought

A drought is an event of prolonged conditions with a well below-average water supply that results in negative impacts on the natural systems and economic sectors. Because of the complexity of impacts, droughts are often not directly measurable, but can be characterized using different indicators. Different drought indices such as the Standardized Precipitation Index, Standardized Precipitation-Evapotranspiration Index and the Palmer Drought Severity Index, have been used to study past changes in droughts for different parts of China, making it difficult to intercompare and synthesize results across the studies. Attribution studies are limited to attributing specific drought events, finding an increase in the probability of the drought events as a result of human influence, including the autumn drought of 2009 in southwestern China [107] and the late spring drought of 2018 in South China [108]. Li *et al.* [109] found detectable human contribution to the intensification of summer hot drought events in northeastern China where drought events were loosely defined as high temperature and low precipitation through a joint probability distribution of precipitation and temperature. As drought events are defined based on the joint probability distribution, it is difficult to compare those events with droughts that are defined based on traditional drought indicators. Overall, while there is increased attention in attributing droughts, there is still a lack of general understanding of the human influence on droughts in China.

## CONCLUSION AND FUTURE DEVELOPMENT

Despite entering the fields of climate change detection and attribution and event attribution relatively late, Chinese researchers have made significant progress over recent years. These studies have established clear and robust evidence of the effects of human influence on mean and extreme temperatures, and emerging evidence for the effects of human influence on extreme precipitation (Box 1). However, a significant gap remains in our understanding of how human influence affects other aspects of climate change and climate impacts in China.

**Box 1.** Summary of attribution of human influence on changes in China's climate since the mid-20th century.

Changes in long-term mean	
**Climate variables or phenomenon**	**Attribution of human influence**
Mean temperature	Very likely the main driver for the observed increase
Total precipitation	Lack of change in observations
Frequency and intensity of hot extremes	Very likely the main driver for the observed increase
Frequency and intensity of cold extremes	Very likely the main driver for the observed decrease
Frequency and intensity of heavy precipitation	Human influence for an increase in heavy precipitation emerging
Drought	Limited evidence for increase in droughts
Extreme events	
**Type of events**	**Attribution of human influence**
Extreme heat	Very likely increase in occurrence probability
Extreme cold	Very likely decrease in occurrence probability
Heavy precipitation	Mixed signal (increase in probability for some events but decrease for other events)

**Figure 7.**
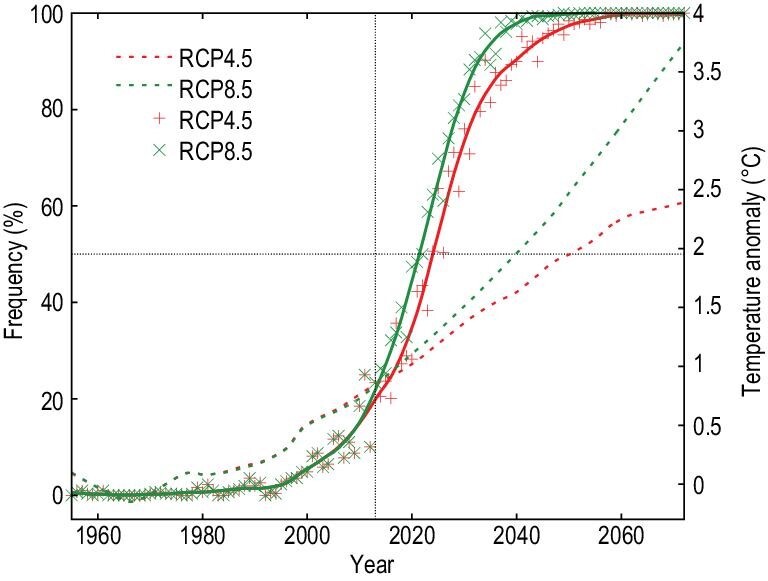
Frequency of 2013-like summer increases rapidly. The figure shows time evolution (solid lines) of the frequency for summer temperature anomalies (relative to the 1955–1984 mean) that are above 1.1°C in observation constrained projection under RCP4.5 and RCP8.5 scenarios (left-hand scale). The solid smooth curves show LOESS (local regression) fitting. For reference, the dashed curves show the projected changes in mean temperature (right-hand scale) under RCP4.5 and RCP8.5 scenarios. Adapted with permission from Ref. [51]. Copyright 2014 Nature Publishing Group.

Human influence on the temperature in China has now been studied extensively. The impact of human activities on temperature is clear, regardless of differences in the metrics including long-term changes in mean or extreme temperatures, and the frequency and magnitude of events of temperature extremes. The effects are also clear, irrespective of differences in the methods used for the analyses. Anthropogenic forcings, dominated by GHG emissions, are the main driver for the increases in mean and extreme temperatures that have occurred since the 1960s. The cooling effects from other anthropogenic forcings including aerosols partially offset the GHG-induced warming. But precisely separating the cooling effect of aerosol from the GHG’s warming effect on China's temperature is difficult because these effects are highly colinear. Urbanization also significantly contributed to warming, especially at a local level. Together, these have led to an increase in hot extremes and a decrease in cold extremes: hot extremes have become more frequent, more intense and longer in duration, while cold extremes have become less frequent and less intense. For example, the 2013 summer heatwave in eastern China would have been extremely unlikely without the effects of human influence, and it has now become a one-in-four to one-in-five year event. This has clear implications for future changes in extreme high-temperature events. The observation-based future projection shows that the 2013-like heatwave will become a once-a-year event around the 2050s, even under a medium emission scenario (Fig. 7). This conclusion is confirmed by another independent study, which used large-ensemble runs and a model-bias adjustment method [110].

There is not clear evidence of anthropogenic influence on total precipitation, although some numerical experiments have shown the effects of aerosols. The evidence of human influence on extreme precipitation is emerging, however. This includes detection of an anthropogenic signal in long-term changes to the magnitude and frequency of heavy precipitation, although the detection is not very robust because of variations in the methods used for data processing and analysis. These findings also project an increased magnitude or frequency for some extreme precipitation events. The effects of human influence on individual heavy precipitation events are still uncertain. Conclusions vary from study to study, which suggests that the effect of human activities may either increase or decrease the occurrence of heavy precipitation in different regions or scenarios.

Human influence on other climate variables or climate extremes has not been widely studied, such as wind, extreme wind, typhoon and climate impact relevant variables. Among these variables and events, change in mean wind speed has been fairly extensively detected [59,111–113], and the wind variability and change have started to be attributed to factors such as increased surface roughness [114] and anthropogenic warming [115]. The changes in circulation, such as the weakening and widening of the Hadley circulation [116], would also have important influence on regional climate change in China. End-to-end event attribution [37], which attributes the impacts from climate events to human influence, is still lacking. There is also a need to understand, and to reduce the uncertainty for attribution, and event attribution in particular. Significant work is required to meet climate adaptation and mitigation policymaking needs. Identification of the effects of climate change mitigations will be another significant challenge.

## References

[1] IPCC . Summary for policymakers. In: Masson-Delmotte V, Zhai P, Pörtner H-O et al. (eds.). Global Warming of 1.5°C. Cambridge and New York: Cambridge University Press, 2018, 1–24.

[2] CMA Climate Change Center : Blue Book on Climate Change in China (2020). Beijing: Science Press, 2020.

[3] IPCC . Climate change. In: Houghton JT, Jenkins GJ, Ephraums JJ (eds.). The IPCC Scientific Assessment Contribution of Working Group I to the First Assessment Report of the Intergovernmental Panel on Climate Change. Cambridge and New York: Cambridge University Press, 1990.

[4] IPCC . Climate change 1995. In: Houghton JT, Meira Filho LG, Callander BA et al. (eds.). The Science of Climate Change Contribution of Working Group I to the Second Assessment Report of the Intergovernmental Panel on Climate Change. Cambridge and New York: Cambridge University Press, 1996.

[5] IPCC . Climate change 2001. In: Houghton JT, Ding Y, Griggs DJ et al. (eds.). The Scientific Basis Contribution of Working Group I to the Third Assessment Report of the Intergovernmental Panel on Climate Change. Cambridge and New York: Cambridge University Press, 2001.

[6] IPCC . Climate change 2007. In: Solomon S, Qin D, Manning M et al. (eds.). The Physical Science Basis Contribution of Working Group I to the Fourth Assessment Report of the Intergovernmental Panel on Climate Change. Cambridge and New York: Cambridge University Press, 2007.

[7] IPCC . Climate change 2013. In: Stocker TF, Qin D, Plattner G-K et al. (eds.). The Physical Science Basis Contribution of Working Group I to the Fifth Assessment Report of the Intergovernmental Panel on Climate Change. Cambridge and New York: Cambridge University Press, 2013.

[8] Committee of Chinese National Assessment Report on Climate Change . The Third China's National Assessment Report on Climate Change (in Chinese). Beijing: China Science Press, 2016.

[9] Qin DH , Dong WJ, Lou Y. Climate and Environment Changes in China (Volume 1) (in Chinese). Beijing: China Meteorological Press, 2012.

[10] Sun Y , Yin H, Tian QH et al. Recent progress in studies of climate change detection and attribution in the globe and China in the past 50 years (in Chinese). Adv Clim Chang Res 2013; 9: 235–54. 10.3969/j.issn.1673-1719.2013.04.001

[11] Bindoff NL , Stott PA, AchutaRao KM et al. Detection and attribution of climate change: from global to regional. In: Change IPoC (ed.) Climate Change 2013: the Physical Science Basis. Working Group I Contribution to the Fifth Assessment Report of the Intergovernmental Panel on Climate Change. Cambridge: Cambridge University Press, 2013, 867–952.

[12] Stott PA , Christidis N, Otto FE et al. Attribution of extreme weather and climate-related events. WIREs Clim Chang 2016; 7: 23–41. 10.1002/wcc.380 PMC4739554 26877771

[13] Zhai P , Zhou B, Chen Y. A review of climate change attribution studies. J Meteorol Res 2018; 32: 671–92. 10.1007/s13351-018-8041-6

[14] Allen MR , Stott PA. Estimating signal amplitudes in optimal fingerprinting, part I: theory. Clim Dyn 2003; 21: 477–91. 10.1007/s00382-003-0313-9

[15] Ribes A , Planton S, Terray L. Application of regularised optimal fingerprinting to attribution. Part I: method, properties and idealised analysis. Clim Dyn 2013; 41: 2817–36. 10.1007/s00382-013-1735-7

[16] Ribes A , Terray L. Application of regularised optimal fingerprinting to attribution. Part II: application to global near-surface temperature. Clim Dyn 2013; 41: 2837–53. 10.1007/s00382-013-1736-6

[17] Ribes A , Zwiers FW, Azais J-M et al. A new statistical approach to climate change detection and attribution. Clim Dyn 2017; 48: 367–86. 10.1007/s00382-016-3079-6

[18] Hasselmann KF. On the signal-to-noise problem in atmospheric response studies. In: Joint Conference of Royal Meteorological Society, American Meteorological Society, Deutsche Meteorologische Gesellschaft and the Royal Society. Royal Meteorol Soc, 1979, 251–9.

[19] Allen MR , Tett SFB. Checking for model consistency in optimal fingerprinting. Clim Dyn 1999; 15: 419–34. 10.1007/s003820050291

[20] Hannart A , Ribes A, Naveau P. Optimal fingerprinting under multiple sources of uncertainty. Geophys Res Lett 2014; 41: 1261–8. 10.1002/2013GL058653

[21] Katzfuss M , Hammerling D, Smith RL. A Bayesian hierarchical model for climate change detection and attribution. Geophys Res Lett 2017; 44: 5720–8. 10.1002/2017GL073688

[22] DelSole T , Trenary L, Yan XQ et al. Confidence intervals in optimal fingerprinting. Clim Dyn 2019; 52: 4111–26. 10.1007/s00382-018-4356-3

[23] Min SK , Zhang X, Zwiers FW et al. Human contribution to more-intense precipitation extremes. Nature 2011; 470: 378–81. 10.1038/nature09763 21331039

[24] Min S-K , Zhang X, Zwiers F et al. Multimodel detection and attribution of extreme temperature changes. J Clim 2013; 26: 7430–51. 10.1175/JCLI-D-12-00551.1

[25] Zhang X , Wan H, Zwiers FW et al. Attributing intensification of precipitation extremes to human influence. Geophys Res Lett 2013; 40: 5252–7. 10.1002/grl.51010

[26] Kim Y-H , Min S-K, Zhang X et al. Attribution of extreme temperature changes during 1951–2010. Clim Dyn 2016; 46: 1769–82. 10.1007/s00382-015-2674-2

[27] Seong M-G , Min S-K, Kim Y-H et al. Anthropogenic greenhouse gas and aerosol contributions to extreme temperature changes during 1951–2015. J Clim 2021; 34: 857–70. 10.1175/JCLI-D-19-1023.1

[28] Kirchmeier-Young MC , Zhang X. Human influence has intensified extreme precipitation in North America. Proc Natl Acad Sci USA 2020; 117: 13308–13. 10.1073/pnas.1921628117 32482861 PMC7306817

[29] Wen QH , Zhang X, Xu Y et al. Detecting human influence on extreme temperatures in China. Geophys Res Lett 2013; 40: 1171–6. 10.1002/grl.50285

[30] Zwiers FW , Zhang X, Feng Y. Anthropogenic influence on long return period daily temperature extremes at regional scales. J Clim 2011; 24: 881–92. 10.1175/2010JCLI3908.1

[31] Wang J , Tett SFB, Yan Z. Correcting urban bias in large-scale temperature records in China, 1980–2009. Geophys Res Lett 2017; 44: 401–8. 10.1002/2016GL071524

[32] Wang J , Chen Y, Tett SF et al. Anthropogenically-driven increases in the risks of summertime compound hot extremes. Nat Commun 2020; 11: 528. 10.1038/s41467-019-14233-8 32047147 PMC7012878

[33] Stott PA , Stone DA, Allen MR. Human contribution to the European heatwave of 2003. Nature 2004; 432: 610–4. 10.1038/nature03089 15577907

[34] Peterson TC , Stott PA, Herring S. Explaining extreme events of 2011 from a climate perspective. Bull Amer Meteor Soc 2012; 93: 1041–67. 10.1175/BAMS-D-12-00021.1

[35] National Academies of Sciences, Engineering and Medicine . Attribution of Extreme Weather Events in the Context of Climate Change: Washington: National Academies Press, 2016.

[36] Hope P , Wang G, Lim E-P et al. What caused the record-breaking heat across Australia in October 2015? Bull Am Meteorol Soc 2016; 97: S122–6. 10.1175/BAMS-D-16-0141.1

[37] Otto FEL. Attribution of weather and climate events. Annu Rev Environ Resour 2017; 42: 627–46. 10.1146/annurev-environ-102016-060847

[38] Shepherd TG , Boyd E, Calel RA et al. Storylines: an alternative approach to representing uncertainty in physical aspects of climate change. Clim Chang 2018; 151: 555–71. 10.1007/s10584-018-2317-9 PMC6394420 30880852

[39] Angélil O , Stone D, Wehner M et al. An independent assessment of anthropogenic attribution statements for recent extreme temperature and rainfall events. J Clim 2017; 30: 5–16. 10.1175/JCLI-D-16-0077.1

[40] Li C , Sun Y, Zwiers F et al. Rapid warming in summer wet bulb globe temperature in China with human-induced climate change. J Clim 2020; 33: 5697–711. 10.1175/JCLI-D-19-0492.1

[41] Meehl GA , Covey C, Delworth T et al. The WCRP CMIP3 multimodel dataset: a new era in climate change research. Bull Am Meteorol Soc 2007; 88: 1383–94. 10.1175/BAMS-88-9-1383

[42] Taylor KE , Stouffer RJ, Meehl GA. An overview of CMIP5 and the experiment design. Bull Am Meteorol Soc 2012; 93: 485–98. 10.1175/BAMS-D-11-00094.1

[43] Eyring V , Bony S, Meehl GA et al. Overview of the coupled model intercomparison project phase 6 (CMIP6) experimental design and organization. Geosci Model Dev 2016; 9: 1937–58. 10.5194/gmd-9-1937-2016

[44] Zhang X , Zwiers FW, Stott PA. Multimodel multisignal climate change detection at regional scale. J Clim 2006; 19: 4294–307. 10.1175/JCLI3851.1

[45] Xu Y , Gao X, Shi Y et al. Detection and attribution analysis of annual mean temperature changes in China. Clim Res 2015; 63: 61–71. 10.3354/cr01283

[46] Sun Y , Zhang X, Ren G et al. Contribution of urbanization to warming in China. Nat Clim Chang 2016; 6: 706–9. 10.1038/nclimate2956

[47] Santer BD , Bonfils C, Painter JF et al. Volcanic contribution to decadal changes in tropospheric temperature. Nat Geosci 2014; 7: 185–9. 10.1038/ngeo2098

[48] Ren G , Zhou Y. Urbanization effect on trends of extreme temperature indices of national stations over mainland China, 1961–2008. J Clim 2014; 27: 2340–60. 10.1175/JCLI-D-13-00393.1

[49] Yan Z-W , Wang J, Xia J-J et al. Review of recent studies of the climatic effects of urbanization in China. Adv Clim Chang Res 2016; 7: 154–68. 10.1016/j.accre.2016.09.003

[50] Zhao T , Li C, Zuo Z. Contributions of anthropogenic and external natural forcings to climate changes over China based on CMIP5 model simulations. Sci China Earth Sci 2016; 59: 503–17. 10.1007/s11430-015-5207-2

[51] Sun Y , Zhang X, Zwiers FW et al. Rapid increase in the risk of extreme summer heat in eastern China. Nat Clim Chang 2014; 4: 1082–5. 10.1038/nclimate2410

[52] Wang Y , Sun Y, Hu T et al. Attribution of temperature changes in western China. Int J Climatol 2018; 38: 742–50. 10.1002/joc.5206

[53] IPCC . Climate change 2013. In: Stocker TF, Qin D and Plattner G-K *et al*. (eds.). The Physical Science Basis Contribution of Working Group I to the Fifth Assessment Report of the Intergovernmental Panel on Climate Change. Cambridge and New York: Cambridge University Press, 2013.

[54] Ding Y , Wang Z, Sun Y. Inter-decadal variation of the summer precipitation in east China and its association with decreasing Asian summer monsoon. Part I: observed evidences. Int J Climatol 2008; 28: 1139–61. 10.1002/joc.1615

[55] Ding YH , Sun Y, Wang ZY et al. Inter-decadal variation of the summer precipitation in China and its association with decreasing Asian summer monsoon part II: possible causes. Int J Climatol 2009; 29: 1926–44. 10.1002/joc.1759

[56] Zhang X , Zwiers FW, Hegerl GC et al. Detection of human influence on twentieth-century precipitation trends. Nature 2007; 448: 461–5. 10.1038/nature06025 17646832

[57] Jiang M , Li Z, Wan B et al. Impact of aerosols on precipitation from deep convective clouds in eastern China. J Geophys Res Atmos 2016; 121: 9607–20. 10.1002/2015JD024246 PMC7430198 32818125

[58] Tian F , Dong B, Robson J et al. Forced decadal changes in the east Asian summer monsoon: the roles of greenhouse gases and anthropogenic aerosols. Clim Dyn 2018; 51: 3699–715. 10.1007/s00382-018-4105-7

[59] Zhang J , Zhao T, Dai A et al. Detection and attribution of atmospheric precipitable water changes since the 1970s over China. Sci Rep 2019; 9: 17609. 10.1038/s41598-019-54185-z 31772341 PMC6879575

[60] Zhou B , Xu Y, Wu J et al. Changes in temperature and precipitation extreme indices over China: analysis of a high-resolution grid dataset. Int J Climatol 2016; 36: 1051–66. 10.1002/joc.4400

[61] Yin H , Sun Y, Wan H et al. Detection of anthropogenic influence on the intensity of extreme temperatures in China. Int J Climatol 2017; 37: 1229–37. 10.1002/joc.4771

[62] Lu C , Sun Y, Wan H et al. Anthropogenic influence on the frequency of extreme temperatures in China. Geophys Res Lett 2016; 43: 6511–8. 10.1002/2016GL069296

[63] Lu C , Sun Y, Zhang X. Multimodel detection and attribution of changes in warm and cold spell durations. Environ Res Lett 2018; 13: 074013. 10.1088/1748-9326/aacb3e

[64] Yin H , Sun Y. Detection of anthropogenic influence on fixed threshold indices of extreme temperature. J Clim 2018; 31: 6341–52. 10.1175/JCLI-D-17-0853.1

[65] Hu T , Sun Y, Zhang XB et al. Human influence on frequency of temperature extremes. Environ Res Lett 2020; 15: 064014. 10.1088/1748-9326/ab8497

[66] Dunn RJH , Alexander LV, Donat MG et al. Development of an updated global land in-situ-based dataset of temperature and precipitation extremes: HadEX3. J Geophys Res Atmos 2020: e2019JD032263 10.1029/2019JD032263.

[67] Sun Y , Hu T, Zhang X et al. Contribution of global warming and urbanization to changes in temperature extremes in eastern China. Geophys Res Lett 2019; 46: 11426–34. 10.1029/2019GL084281

[68] Yin H , Sun Y, Donat MG. Changes in temperature extremes on the Tibetan Plateau and their attribution. Environ Res Lett 2019; 14: 124015. 10.1088/1748-9326/ab503c

[69] Fu JL , Qian WH, Lin X et al. Trends in graded precipitation in China from 1961 to 2000. Adv Atmos Sci 2008; 25: 267–78. 10.1007/s00376-008-0267-2

[70] Liu R , Liu SC, Cicerone RJ et al. Trends of extreme precipitation in eastern China and their possible causes. Adv Atmos Sci 2015; 32: 1027–37. 10.1007/s00376-015-5002-1

[71] Shi PJ , Bai XM, Kong F et al. Urbanization and air quality as major drivers of altered spatiotemporal patterns of heavy rainfall in China. Lands Ecol 2017; 32: 1723–38. 10.1007/s10980-017-0538-3

[72] Qian Y , Gong D, Fan J et al. Heavy pollution suppresses light rain in China: observations and modeling. J Geophys Res Atmos 2009; 114: D00K02. 10.1029/2008JD011575

[73] Liang P , Ding YH. The long-term variation of extreme heavy precipitation and its link to urbanization effects in Shanghai during 1916–2014. Adv Atmos Sci 2017; 34: 321–34. 10.1007/s00376-016-6120-0

[74] Zhai PM , Zhang XB, Wan H et al. Trends in total precipitation and frequency of daily precipitation extremes over China. J Clim 2005; 18: 1096–108. 10.1175/JCLI-3318.1

[75] Yin H , Sun Y. Characteristics of extreme temperature and precipitation in China in 2017 based on ETCCDI indices. Adv Clim Chang Res 2018; 9: 218–26. 10.1016/j.accre.2019.01.001

[76] Li H , Chen H, Wang H. Effects of anthropogenic activity emerging as intensified extreme precipitation over China. J Geophys Res Atmos 2017; 122: 6899–914. 10.1002/2016JD026251

[77] Li W , Jiang Z, Zhang X et al. On the emergence of anthropogenic signal in extreme precipitation change over China. Geophys Res Lett 2018; 45: 9179–85. 10.1029/2018GL079133

[78] Ma S , Zhou T, Stone DA et al. Detectable anthropogenic shift toward heavy precipitation over eastern China. J Clim 2017; 30: 1381–96. 10.1175/JCLI-D-16-0311.1

[79] Chen Y , Li W, Jiang X et al. Detectable intensification of hourly-and daily-scale precipitation extremes across eastern China. J Clim 2020; 34: 1185–201. 10.1175/JCLI-D-20-0462.1

[80] Chen H , Sun J. Contribution of human influence to increased daily precipitation extremes over China. Geophys Res Lett 2017; 44: 2436–44. 10.1002/2016GL072439

[81] Zhang X , Alexander L, Hegerl GC et al. Indices for monitoring changes in extremes based on daily temperature and precipitation data. WIREs: Clim Change 2011; 2: 851–70. 10.1002/wcc.147

[82] Dong S , Sun Y, Li C. Detection of human influence on precipitation extremes in Asia. J Clim 2020; 33: 5293–304. 10.1175/JCLI-D-19-0371.1

[83] Dong S , Sun Y, Li C et al. Attribution of extreme precipitation with updated observations and CMIP6 simulations. J Clim 2021; 34: 871–81. 10.1175/JCLI-D-19-1017.1

[84] Song L , Dong S, Sun Y et al. Role of anthropogenic forcing in 2014 hot spring in northern China. Bull Am Meteorol Soc 2015; 96: S111–4. 10.1175/BAMS-D-15-00111.1

[85] Chen Y , Chen W, Su Q et al. Anthropogenic warming has substantially increased the likelihood of July 2017-like heat waves over central eastern China. Bull Am Meteorol Soc 2019; 100: S91–5. 10.1175/BAMS-D-18-0087.1

[86] Sun Y , Song L, Yin H et al. Human influence on the 2015 extreme high temperature events in western China. Bull Am Meteorol Soc 2016; 97: S102–6. 10.1175/BAMS-D-16-0158.1

[87] Ren L , Wang D, An N et al. Anthropogenic influences on the persistent night-time heat wave in summer 2018 over northeast China. Bull Am Meteorol Soc 2020; 101: S83–8. 10.1175/BAMS-D-19-0152.1

[88] Lu CH , Sun Y, Christidis N et al. Contribution of global warming and atmospheric circulation to the hottest spring in eastern China in 2018. Adv Atmos Sci 2020; 37: 1285–94. 10.1007/s00376-020-0088-5

[89] Jian Y , Lin X, Zhou W et al. Analysis of record-high temperature over southeast coastal China in winter 2018/19: the combined effect of mid-to high-latitude circulation systems and SST forcing over the north Atlantic and tropical western Pacific. J Clim 2020; 33: 8813–31. 10.1175/JCLI-D-19-0732.1

[90] Zhou C , Chen D, Wang K et al. Conditional attribution of the 2018 summer extreme heat over northeast China: roles of urbanization, global warming, and warming-induced circulation changes. Bull Am Meteorol Soc 2020; 101: S71–6. 10.1175/BAMS-D-19-0197.1

[91] Miao C , Sun Q, Kong D et al. Record-breaking heat in northwest China in July 2015: analysis of the severity and underlying causes. Bull Am Meteorol Soc 2016; 97: S97–101. 10.1175/BAMS-D-16-0142.1

[92] Ciavarella A , Christidis N, Andrews M et al. Upgrade of the HadGEM3-A based attribution system to high resolution and a new validation framework for probabilistic event attribution. Weather Clim Extremes 2018; 20: 9–32. 10.1016/j.wace.2018.03.003

[93] Sun Y , Dong S, Hu T et al. Attribution of the warmest spring of 2018 in northeastern Asia using simulations of a coupled and an atmospheric model. Bull Am Meteorol Soc 2020; 101: S129–34. 10.1175/BAMS-D-19-0264.1

[94] Sun Y , Hu T, Zhang XB et al. Anthropogenic influence on the eastern China 2016 super cold surge. Bull Am Meteorol Soc 2018; 99: S123–7. 10.1175/BAMS-D-17-0092.1

[95] Qian C , Wang J, Dong S et al. Human influence on the record-breaking cold event in January of 2016 in eastern China. Bull Am Meteorol Soc 2018; 99: S118–22. 10.1175/BAMS-D-17-0095.1

[96] Ma S , Zhu C. Extreme cold wave over east Asia in January 2016: a possible response to the larger internal atmospheric variability induced by arctic warming. J Clim 2019; 32: 1203–16. 10.1175/JCLI-D-18-0234.1

[97] Otto FEL , Massey N, van Oldenborgh GJ et al. Reconciling two approaches to attribution of the 2010 Russian heat wave. Geophys Res Lett 2012; 39: L04702. 10.1029/2011GL050422

[98] Sun QH , Miao CY. Extreme rainfall (R20mm, Rx5day) in Yangtze-Huai, China, in June-July 2016: the role of ENSO and anthropogenic climate change. Bull Am Meteorol Soc 2018; 99: S102–6. 10.1175/BAMS-D-17-0091.1

[99] Yuan X , Wang S, Hu Z-Z. Do climate change and El Niño increase likelihood of Yangtze River extreme rainfall? Bull Am Meteorol Soc 2018; 99: S113–7. 10.1175/BAMS-D-17-0089.1

[100] Sun Y , Dong S, Hu T et al. Anthropogenic influence on the heaviest June precipitation in southeastern China since 1961. Bull Am Meteorol Soc 2019; 100: S79–83. 10.1175/BAMS-D-18-0114.1

[101] Burke C , Stott P, Sun Y et al. Attribution of extreme rainfall in southeast China during May 2015. Bull Am Meteorol Soc 2016; 97: S92–6. 10.1175/BAMS-D-16-0144.1

[102] Li C , Tian Q, Yu R et al. Attribution of extreme precipitation in the lower reaches of the Yangtze River during May 2016. Environ Res Lett 2018; 13: 014015. 10.1088/1748-9326/aa9691

[103] Zhang W , Li W, Zhu L et al. Anthropogenic influence on 2018 summer persistent heavy rainfall in central western China. Bull Am Meteorol Soc 2020; 101: S65–70. 10.1175/BAMS-D-19-0147.1

[104] Zhou C , Wang K, Qi D. Attribution of the July 2016 extreme precipitation event over China's Wuhang. Bull Am Meteorol Soc 2018; 99: S107–12. 10.1175/BAMS-D-17-0090.1

[105] Zhou B , Wang Z, Shi Y et al. Historical and future changes of snowfall events in China under a warming background. J Clim 2018; 31: 5873–89. 10.1175/JCLI-D-17-0428.1

[106] Zhou B , Wang Z, Sun B et al. Decadal change of heavy snowfall over northern China in the mid-1990s and associated background circulations. J Clim 2021; 34: 825–37. 10.1175/JCLI-D-19-0815.1

[107] Ma S , Zhou T, Angélil O et al. Increased chances of drought in southeastern periphery of the Tibetan Plateau induced by anthropogenic warming. J Clim 2017; 30: 6543–60. 10.1175/JCLI-D-16-0636.1

[108] Zhang L , Zhou T, Chen X et al. The late spring drought of 2018 in south China. Bull Am Meteorol Soc 2020; 101: S59–64. 10.1175/BAMS-D-19-0202.1

[109] Li H , Chen H, Sun B et al. A detectable anthropogenic shift toward intensified summer hot drought events over northeastern China. Earth and Space Sci 2020; 7: e2019EA000836. 10.1029/2019EA000836

[110] Sun Y , Hu T, Zhang X. Substantial increase in heat wave risks in China in a future warmer world. Earth's Future 2018; 6: 1528–38. 10.1029/2018EF000963

[111] Chen L , Li D, Pryor SC. Wind speed trends over China: quantifying the magnitude and assessing causality. Int J Climatol 2013; 33: 2579–90. 10.1002/joc.3613

[112] Zhang G , Azorin-Molina C, Chen D et al. Variability of daily maximum wind speed across China, 1975–2016: an examination of likely causes. J Clim 2020; 33: 2793–816. 10.1175/JCLI-D-19-0603.1

[113] Lin C , Yang K, Huang J et al. Impacts of wind stilling on solar radiation variability in China. Sci Rep 2015; 5: 15135. 10.1038/srep15135 26463748 PMC4604519

[114] Zhang ZT , Wang KC, Chen DL et al. Increase in surface friction dominates the observed surface wind speed decline during 1973–2014 in the northern hemisphere lands. J Clim 2019; 32: 7421–35. 10.1175/JCLI-D-18-0691.1

[115] Zhang G , Azorin-Molina C, Chen D et al. Uneven warming likely contributed to declining wind speeds in northern China between 1961 and 2016. J Geophys Res Atmos 2021; e2020JD033637. 10.1029/2020JD033637

[116] Xia Y , Hu Y, Liu J. Comparison of trends in the Hadley circulation between CMIP6 and CMIP5. Sci Bull 2020; 65: 1667–74. 10.1016/j.scib.2020.06.011 36659043

